# Influence of applying different units of measurement on reporting antimicrobial consumption data for pig farms

**DOI:** 10.1186/s12917-015-0566-7

**Published:** 2015-10-06

**Authors:** FJ Taverne, JH Jacobs, DJJ Heederik, JW Mouton, JA Wagenaar, IM van Geijlswijk, on behalf of the Netherlands Veterinary Medicines Authority (SDa)

**Affiliations:** The Netherlands Veterinary Medicines Authority (SDa), Yalelaan 114, 3584 CM Utrecht, The Netherlands; Institute for Risk Assessment Sciences, Utrecht University, Yalelaan 2, 3584 CM Utrecht, The Netherlands; Pharmacy Department, Faculty of Veterinary Medicine, Utrecht University, Yalelaan 106, 3584 CM Utrecht, The Netherlands; Department of Medical Microbiology and Infectious Diseases, Erasmus MC, Wytemaweg 80, 3015 CN Rotterdam, The Netherlands; Department of Infectious Diseases & Immunology, Faculty of Veterinary Medicine, Utrecht University, Yalelaan 1, 3584 CL Utrecht, The Netherlands; Central Veterinary Institute, Wageningen UR, Houtribweg 39, 8221 RA Lelystad, The Netherlands

**Keywords:** Methodology, International, Antimicrobial consumption monitoring, Livestock, Daily dosages, Veterinary medicine

## Abstract

**Background:**

Antimicrobial use in livestock is one of the factors contributing to selection and spread of resistant microorganisms in the environment. National veterinary antimicrobial consumption monitoring programs are therefore in place in a number of countries in the European Union. However, due to differences in methodology, results on veterinary antimicrobial consumption from these national monitoring programs cannot be compared internationally. International comparison is highly needed to establish regulations on veterinary antimicrobial use and reducing antimicrobial resistance. The aim of this study was to assess differences in the outcomes on veterinary antimicrobial consumption by applying three different sets of nationally established animal defined daily dosages to the same antimicrobial drug delivery dataset of Dutch pigs in 2012.

**Methods:**

Delivery information for the complete Dutch pig sector for the year 2012 reported to the Netherlands Veterinary Medicines Authority (SDa) was analysed with three differently and nationally established animal defined daily dosages from the Netherlands and Denmark: the Defined Daily Dosage Animal_NL_ (DDDA_NL_), the Animal Daily Dosage_DK_ (ADD_DK_) and Defined Animal Daily Dosage_DK_ (DADD_DK_). For each applied Dutch product equivalent, Danish products were identified based on authorization for pigs, active substance (including form), administration route, concentration and dosage regimen.

**Results:**

Consumption in number of ADD_DK_/Y was lower than in number of DDDA_NL_/Y for sows/piglets and finisher pigs, with proportions of 83.3 % and 98.3 %. Use in number of DADD_DK_/Y was even lower, 79.7 % for sows/piglets and 88.1 % for finisher pigs compared to number of DDDA_NL_/Y. At therapeutic group level proportions of number of DADD_DK_/Y to number of DDDA_NL_/Y were 63.6-150.4 % (sows/piglets) and 55.6-171.0 % (finisher pigs). Proportions were > 100 % for the polymyxines (sows/piglets 150.4 % and finisher pigs 149.9 %) and the macrolides/lincosamides (finisher pigs 171.0 %).

**Conclusions:**

Differences between nationally established animal defined daily dosages caused by different correction factors for long-acting products and national differences in authorized dosages, have a substantial influence on the results of antimicrobial consumption in pigs. To enable international comparison of veterinary antimicrobial consumption data, harmonized units of measurement, animal weights and animal (sub) categories are needed.

## Background

Antimicrobial Veterinary Medicinal Product (AVMP) use in livestock is one of the main factors contributing to selection and spread of resistant microorganisms, such as extended-spectrum beta-lactamase producing microorganisms (ESBLs) [1]. The animals and their environment form a reservoir for the transmission of resistant pathogens to other animals and potentially to humans [2–6]. To minimize selection and spread of antimicrobial resistance both at national and international levels, the antimicrobial burden needs to be reduced [7] and more prudent use of antimicrobials in human and veterinary medicine is necessary.

Some European countries have implemented a national system for monitoring antimicrobial consumption by humans and animals and publish the results in national reports, such as NethMap [8], MARAN [9] and the SDa report [10] in the Netherlands. Sales data are used to monitor veterinary antimicrobial use, but some national monitoring systems also use animal defined daily dosages as their unit of measurement. The European Medicines Agency reports sales data of veterinary antimicrobials per country through the European Surveillance of Veterinary Antimicrobial Consumption (ESVAC) project [11]. A working party of the ESVAC aims to specify the reports for example by stratifying sales of specific AVMPs to animal (sub) categories [12] and to establish internationally harmonized animal defined daily dosages, comparable to the units used in the Netherlands and Denmark [10, 13] in order to enable cross country comparison. In human medicine internationally harmonized Defined Daily Dosages (DDDs) per active ingredient are established by the WHO [14]. DDDs are established for the main indication for which the active ingredient is applied and corrects for differences in dosing between various active ingredients for the same indication . Because DDDs are internationally applied in reporting medicine use, the results in these reports are suitable for direct international comparison. Animal defined daily doses, however, are currently only established at a national level. The principle of the animal defined daily dosage is to establish a standard dose per product, per animal species, per day to correct for differences in dosing between antimicrobials and animal species. Therefore, the principle of this unit of measurement for monitoring of antimicrobial consumption is the same in the different countries where it is applied. However, due to national product availability, legal considerations such as differences in authorized indications and dosages (since authorization of medicinal products still occurs mainly on a national rather than on an European level) differences in animal defined daily dosages applied in different countries can be expected. This lack of uniformity complicates international comparison of the results from national veterinary antimicrobial drug consumption monitoring programs.

Postma et al. [15] recently presented a concept for establishing internationally harmonized animal defined daily dosages, based on the national authorization documents of individual AVMPs in multiple countries, and described the benefits and limitations of this method. A comparison and review of possible differences between nationally established animal defined daily dosages, however, has not yet been performed. The objective of this study was to compare the animal defined daily dosages applied (currently or in the past) in national veterinary antimicrobial drug consumption monitoring programmes of two countries, to determine whether there are differences and, if differences exist, to analyse the magnitude of these differences. We did this by reviewing the consequences on the outcome on antimicrobial consumption when different animal defined daily dosages were applied to the same dataset of antimicrobial drug deliveries to Dutch pig farms.

## Methods

### Animal defined daily dosages

The defined daily dose animal (DDDA) is the unit of measurement for the determination of annual veterinary antimicrobial drug consumption. In the Netherlands, DDDA’s are derived from Dutch AVMP authorization documents and we will refer to this parameter as the DDDA_NL_. The DDDA_NL_ is established per AVMP and species and represents the amount of AVMP (in g or mL or pieces) needed for the treatment of one kg of animal for one day in accordance to the mean authorized dosage for the target animal. The DDDA_NL_ for AVMPs with a prolonged duration of action (i.e. more than 24 h) or a shorter duration of action (applied 2 times daily) are corrected for this by a correction factor either retrieved from the authorization documents or based on expert consensus within the SDa. Full details on DDDA_NL_ definition as well as data collection, exact contents of datasets, and calculation of the total veterinary antimicrobial consumption in livestock were published previously [16, 17].

A comparable parameter for calculation of annual antimicrobial drug consumption is referred to as Animal Daily Dose (ADD_DK_). The ADD_DK_ is the assumed average maintenance dose needed to treat one kg of animal during one day for the main indication in the target species in accordance with the Danish authorization document of the specific AVMP [13, 18]. The ADD_DK_ was applied as the unit of measurement in DANMAP 2009–2011. In the provided data for this study the ADD_DK_ was expressed as the amount of an AVMP (in g or mL or pieces) needed per kg animal per day.

The third applied animal defined daily dosages are the Defined Animal Daily Dose (DADD_DK_) as reported since DANMAP 2012 [19]. The DADD_DK_ is specified per animal species and is a standardized dose in mg/kg per day for the main indication of a given active ingredient and a specified route of administration and pharmaceutical form. For orally administered antimicrobials, a differentiation is made between types of formulations, for example premixed feed or soluble powder for drinking water medication. For some parenterally administered active compounds a differentiation is made between immediate release preparations and those with prolonged effect [20]. Thus, the DADD_DK_ is a measure established at the level of the active ingredient, route of administration and pharmaceutical form and not at the level of a specific AVMP, which are the main differences between the establishment of this unit of measurement and the ADD_DK_ and DDDA_NL_.

### Data collection

Data on deliveries of AVMPs to individual farms for the complete Dutch pig sector from the year 2012 were collected by the quality assurance organisations of the Dutch pig sector, and provided to the Netherlands Veterinary Medicines Authority (SDa) for the monitoring of antimicrobial consumption in livestock. Numbers of animals per farm specified by subcategory (production animals or finisher pigs) and farm types (sows/piglets or finisher pigs) were incorporated in this dataset. Farms that used a closed system housing both sows/piglets and finisher pigs were divided in a sows/piglet farm and a finisher pigs farm. Individual farms were recognizable by an unique number but the encryption key was not available to the researchers, ensuring anonymous analysis of the data.

The SDa provided the database with the DDDA_NL_ per AVMP originally used to process the Dutch pig data of 2012 (version May 2013). A recent list of DDDA_NL_ is publicly available on the SDa website [21]. The Danish ADD_DK_ used in this study were provided by the Danish Ministry of Food, Agriculture and Fisheries. The DADD_DK_ per active compound defined for pigs was retrieved from the DADD description annex of DANMAP 2012 [20] and entered manually into a worksheet.

### Data analysis

**Fig. 1:**
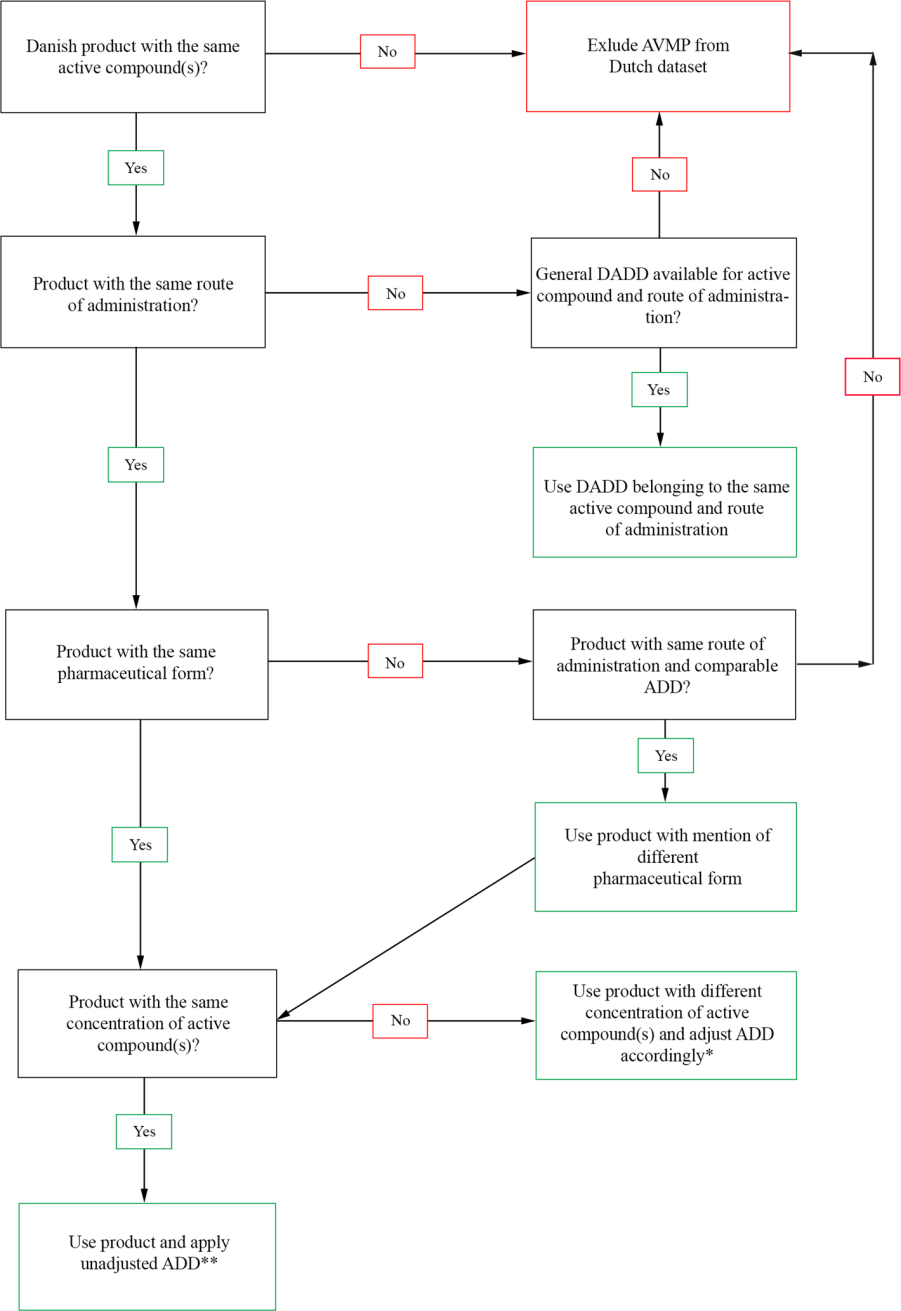
Diagram showing the selection criteria of Danish equivalent products for the Dutch AVMPs. *If the ADD_DK_ of a product with a different concentration correlates better with the DDDA_NL_ then this product is selected instead of the product with a matching concentration of active compound (s). **If more than one product is eligible the product with an ADD_DK_ closest to the DDDA_NL_ is selected

All different AVMPs, identifiable by European Article Number (EAN), delivered to pig farms in the Netherlands in 2012 were derived from the dataset provided by the SDa. Using the ADD_DK_ list from the Danish ministry of Food, Agriculture and Fisheries, Danish equivalent products were selected for each Dutch product. The criteria used in the selection process are shown in Fig. 1. For some products no Danish equivalent could be established, as no products with certain combinations of active compounds or with a certain administration route are authorized in Denmark. Following assessment of the impact of deletion of these records on the number of DDDA_NL_/Y, the delivery records for these products were excluded from all analyses.

Since 2013 the SDa reports the veterinary antimicrobial consumption in livestock as Defined Daily Dosages Animal per year on national level, expressed in this study as the number of DDDA_NL_/Y (identical to the Dutch acronym DDDA_NAT_, but expressed differently here to improve readability of this paper). The total amount of packages including package size (identifiable by EAN) delivered per year per farm type/animal category (sows/piglets or finisher pigs) and the mean numbers of animals at risk per farm type per day were derived from the data provided by the SDa. The standardized animal weights were established by the SDa together with representatives from the Dutch pig sector. The standardized animal weights applied in all analyses in this study are: sows/piglets 303.8 kg (=1 sow (220 kg) + 5.5 piglets (12.5 kg each) + 0.14 gilts (107.5 kg)) and finisher pigs 70 kg.

Using the DDDA_NL_, the delivery records and the package size using the EAN-code, the total amount of kilograms of target animal that can be treated for one day per package of AVMP can be determined. This amount is referred to as amount of treatable kilograms*days. The total amount of treatable kg*days were calculated per animal (sub) category (also referred to as farm type). By dividing this amount with the standardized animal weight for the animal category, the total treatable number of animals*days based on the deliveries was calculated. The number of DDDA_NL_/Y was then calculated per farm type by dividing the total amount of treatable animals*days by the total of animals at risk per day per farm type. The same process was repeated to calculate the number of ADD_DK_/Y and number of DADD_DK_/Y.

Differences between the animal defined daily dosages DDDA_NL_, ADD_DK_ and DADD_DK_ as units of measurement were analysed on a more detailed level by determining the total amounts of treatment days per therapeutic group of AVMPs (as defined by the SDa, based on the ATCvet classification established by the World Health Organization). Sows/piglet farms and finisher pigs farm were analysed separately.

All analyses were performed using SAS® software version 9.2 (SAS institute Inc., Cary, NC, USA).

## Results

The complete Dutch dataset with deliveries of AVMPs to pig farms in 2012 contained 228769 deliveries to a total of 6425 pig farms 2328 of which were breeding farms with sows/piglets and 4628 were farms with finisher pigs. Following the algorithm as displayed in Fig. 1, a total of 13950 deliveries in 2266 farms were excluded from the dataset (6.1 % of total deliveries in the dataset) because there was no Danish equivalent to match the Dutch product. For the sows/piglet farms two farms were excluded from the dataset (2326 farms remaining) after exclusion of these delivery records, for finisher pigs 22 farms were excluded (4606 farms remaining). Of the 13950 excluded records, 12729 records (91 %) were combinations of amoxicillin/colistin, and neomycin/procainbenzylpenicillin, which are combinations of antimicrobials not authorized in Denmark, and trimethoprim/sulfamethoxazole injections (oral trimethoprim/sulfamethoxazole is available in both countries).

Antimicrobial use of Dutch pigs calculated on national level expressed in numbers of DDDA_NL_/Y, ADD_DK_/Y, DADD_DK_/Y are shown in Table 1. Proportions of number of ADD_DK_/Y and number of DADD_DK_/Y to the number of DDDA_NL_/Y were calculated (after exclusion of non-compatible deliveries and with application of Dutch standard animal weights). For sows/piglets the number of ADD_DK_/Y was 83.3 % of the number of DDDA_NL_/Y and the number of DADD_DK_/Y was 79.7 % compared to the number of DDDA_NL_/Y, indicating substantially lower values if calculated by both units used in Denmark. For finisher pigs the proportions were 98.3 % for the number of ADD_DK_/Y and 88.1 % for the number of DADD_DK_/Y to the number of DDDA_NL_/Y.

**Table 1:** Antimicrobial consumption in pigs in the Netherlands in 2012 expressed as numbers of DDDA_NL_/Y, ADD_DK_/Y and DADD_DK_/Y is shown for all delivery records (incl.) and after exclusion of the 13950 delivery records (excl.) that could not be matched to a Danish equivalent

	Farm type	No. of farms	Total no. animals	Total no. of treatment days^b^	Mean^c^	Proportion of reference (%)
DDDA_NL_/Y (excl.)	Sows/piglets	2326	957210^a^	18381559	19.2	Reference
	Finisher pigs	4606	5388248	63476046	11.8	Reference
ADD_DK_/Y	Sows/piglets	2326	957210	15328970	16.0	83.3
	Finisher pigs	4606	5388248	62321913	11.6	98.3
DADD_DK_/Y	Sows/piglets	2326	957210	14658460	15.3	79.7
	Finisher pigs	4606	5388248	56189202	10.4	88.1
DDDA_NL_/Y (incl.)	Sows/piglets	2338	957315	18796613	19.6	102.1
	Finisher pigs	4628	5401503	63786742	11.8	100.0

^a^Only the sows are counted. The piglets and gilts are incorporated in the standardized animal weight. See text for details

^b^Total treatable kilograms*days based on the antimicrobial deliveries per farm type for the whole year 2012. The total treatable kilograms*days were then divided by the standardized animal weight (303.8 kg for sows/piglets and 70 kg for finisher pigs) to calculate total treatment days

^c^The mean was calculated by dividing the total no. of treatable animals based on antimicrobial deliveries by the total number of animals present per farm type

Numbers of DDDA_NL_/Y, ADD_DK_/Y and DADD_DK_/Y per farm type at the level of therapeutic groups were calculated and the results are shown in Table 2. This analysis revealed an overall tendency towards lower numbers of DADD_DK_/Y compared to DDDA_NL_/Y, using the DADD_DK_ compared to the DDDA_NL_ as the unit of measurement. For sows/piglets the proportions of number of DADD_DK_/Y compared to number of DDDA_NL_/Y ranged from 63.6–150.4 % but were <100 % for most therapeutic groups of antimicrobials. Similarly, for the finisher pigs proportions of number of DADD_DK_/Y compared to number of DDDA_NL_/Y ranged from 55.6-171.0 %. A proportion >100 % was found for the polymyxines for both farm types (150.4 % and 149.9 % for sows/piglets and finisher pigs, respectively). The proportion of number of DADD_DK_/Y to number of DDDA_NL_/Y for macrolides/lincosamides was 171.0 % for the finisher pigs only. The proportion calculated for this therapeutic group for sows/piglets was 90.7 %. To determine why the macrolides/lincosamides exceeded 100 % only in finisher pigs we ran a detailed analysis on product level for this antimicrobial group. We found 71.3 % of delivery records for sows/piglets were products containing tulathromycin. For finisher pigs most delivery records were products containing tylosin (89.0 %).

**Table 2:** Antimicrobial consumption in pigs in the Netherlands in 2012 expressed in numbers of DDDA_NL_/Y, ADD_DK_/Y and DADD_DK_/Y per therapeutic group and per farm type and the proportion of DADD_DK_/Y to DDDA_NL_/Y

	DDDA_NL_/Y	ADD_DK_/Y	DADD_DK_/Y	Proportion DADD_DK_/Y to DDDA_NL_/Y^b^ (%)
Sows/piglets farms				
Amphenicols	0.060	0.030	0.060	100.0
Aminoglycosides	0.004	0.003	0.003	76.7
Cephalosporins 3rd/4th gen.	0.001	0.001	0.001	100.0
Combinations	0.290	0.236	0.185	63.6
Fluoroquinolones	0.001	0.001	0.001	87.7
Macrolides/lincosamides	1.776	1.798	1.610	90.7^a^
Penicillins	5.685	3.882	3.580	63.0
Pleuromutilines	0.736	0.513	0.629	85.4
Polymyxines	1.207	1.199	1.815	150.4
Tetracyclines	6.931	6.640	5.778	83.3
Trimethoprim/sulfonamides^c^	2.513	1.710	1.659	66.0
Total	19.20	16.01	15.32	
Finisher pigs farms				
Amphenicols	0.069	0.035	0.069	100.0
Aminoglycosides	-	-	-	-
Cephalosporins 3rd/4th gen.	0.000	0.000	0.000	100.0
Combinations	0.081	0.062	0.051	62.2
Fluoroquinolones	0.000	0.000	0.000	85.6
Macrolides/lincosamides	1.254	2.216	2.144	171.0^a^
Penicillins	1.003	0.736	0.619	61.7
Pleuromutilines	0.099	0.073	0.077	78.0
Polymyxines	0.169	0.167	0.253	149.9
Tetracyclines	7.454	7.320	6.297	84.5
Trimethoprim/sulfonamides^c^	1.651	0.958	0.918	55.6
Total	11.78	11.57	10.43	

^a^A detailed analysis of this group of antimicrobials is performed on product level. See text for details

^b^This calculation is based on the unrounded numbers of DADD_DK_/Y and DDDA_NL_/Y

^c^Trimethoprim/sulphonamides by oral use, applied in combination

## Discussion

In this study we show that application of different nationally established animal defined daily dosages to the same set of antimicrobial delivery data, using the same animal (sub) categories and standardized weight for all analyses, results in substantial differences in the reported overall outcomes on veterinary antimicrobial consumption in pigs. Differences on the outcome were observed at the level of therapeutic groups of AVMPs. Both increases and decreases were observed in treatable kilograms of animals with the application of one set of nationally defined animal daily dosages compared to another.

Differences in several parts of the national process of establishing a defined animal daily dosage can be identified as responsible for the observed differences. One of these factors is the application of a correction factor to the DDDA of long-acting formulations. As mentioned before, the DDDA_NL_ is corrected for prolonged duration of action. For products administered once daily the correction in DDDA_NL_ is factor 1.0. However, the duration of action of the long-acting AVMPs exceed this 24 h and are therefore corrected by a factor exceeding 1.0. In the ADD_DK_ and DADD_DK_ the daily dosage of the AVMP (for ADD_DK_) or active ingredient (for DADD_DK_) was reduced compared to the registered dose to correct for the duration of action. For example, for some long-acting parenteral AVMP’s with oxytetracycline, a correction factor of 3.5 is applied to the DDDA_NL_ (for100 mL of product, containing 200 mg active substance/mL and a dosage of 20 mg/kg the treatable kilograms of animal are ((200/20) * 100)) * 3.5 = 3500 treatable kilograms*days). The ADD_DK_ is 0.1 mL (=20 mg/kg) parenteral oxytetracycline (with the same concentration) but without correction factors and thus one package of 100 mL of this product provides (200/20)*100 = 1000 treatable kilograms*days. Use of long-acting products can therefore theoretically have a substantial influence on the outcome of antimicrobial consumption, especially in sub-analysis or specific (sub) categories of animals/farms. Unfortunately, the duration of action is not always specified by the manufacturer or deducible from pharmacokinetic studies. Therefore the applied correction factors are mostly based on consensus by national experts and differences are therefore inevitable. Postma et al. [15] also describe the lack of information as a problem when establishing DDDAs for long-acting products and the results from this study support this view.

Secondly, there is a difference in establishing the ADD_DK,_ DADD_DK_ or DDDA_NL_ for products with multiple authorized indications and dosages as briefly mentioned in the materials and methods section. The ADD_DK_ and DADD_DK_ are established by the dosage for the main indication (following the definition of the DDDA by the ESVAC and the DDD by the WHO) for which the AVMP or active compound is authorized. For example the main indication of a valnemulin containing product has a dosage of 3–4 mg/kg per day, so the ADD_DK_ is defined at 3.5 mg/kg. The DDDA_NL_ is always established as the mean of the authorized dosages for all authorized indications for the target animal, ranging from 1–1.5 mg/kg to 10–12 mg/kg and therefore is established at 6 mg/kg per day.. The reason for not establishing the DDDA_NL_ on the main indication, is that the indication for which an AVMP is used on a farm is not part of the delivery data provided to the SDa by the animal sectors. Therefore the main indication of an AVMP can only be guessed or assumed, but there are no data to confirm it.

Thirdly, combination products are not always available in all countries, a problem also encountered in this study. Excluding these products from the analysis, as was done in this study, is one option. However, that is only possible when these products account for only a small part of the total antimicrobial consumption. Handling the combination products as two (or more) separate compounds (for example separating TMPS in two compounds, trimethoprim and a sulphonamide) may also lead to over- or underestimation of antimicrobial consumption. Depending on the compound authorized dosages differ between combination therapy with multiple antimicrobials and monotherapy with the separate compounds.

The impact of the differences mentioned above on the outcome were illustrated by an analysis of antimicrobial use per therapeutic group. We showed that for sows/piglets farms there was a reduction in number of DADD_DK_/Y compared to number of DDDA_NL_/Y for the group of macrolides/lincosamides. The same calculations however increased the number of DADD_DK_/Y to the number of DDDA_NL_/Y proportion for the finisher pigs. This indicates deliveries of very specific (different) AVMPs for sows/piglets compared to the finisher pigs. Therefore we performed the analysis on EAN-code (product level) where we found the main compound prescribed for sows/piglets farms was injectable tulathromycin (71.3 % of delivery records) compared to oral tylosin in finisher pigs (89.0 % of delivery records). The difference in proportions of number of DADD_DK_/Y to number of DDDA_NL_/Y between the two categories of animals can be explained (in part) by the fact that a correction factor is applied to the DDDA_NL_ of tulathromycin due to the long duration of action of this compound (duration of action 8 days, daily dosage 2.5/8 = 0.3125 mg/kg). The DADD_DK_ is also corrected for this (daily dosage 1.0 mg/kg, registered dose 2.5 mg/kg once), but the factor applied differs from the Dutch factor. The use of tulathromycin thus results in a higher DDDA_NL_/Y than DADD_DK_/Y.

For tylosin the daily dosage is higher in the Netherlands than in Denmark, resulting in a lower number of DDDA_NL_/Y compared to the number of DADD_DK_/Y. This finding is also in line with the study of Postma et al. [15] as they found an international 10- fold difference between minimum and maximum recommended dosages for tylosin for feed/water medication, which would almost certainly be reflected in a national DDDA if the studied countries had established those.

Apart from the differences between the units of measurement in the national veterinary antimicrobial monitoring programs as reviewed in this study there are other factors in those monitoring systems that complicate international comparison of veterinary antimicrobial use. Firstly, there are differences in the definition of animal (sub) categories and secondly the differences in standardized weights. In the Dutch data no differentiation in antimicrobial deliveries to sows or (weaned) piglets can be made. Therefore, sows and piglets are analysed as one category with a standardized combined weight of 303.8 kg/sow. In Denmark sows do include suckling piglets but weaned piglets are a separate category and sows and weaned piglets have their own yellow card category [22]. Also, the standardized weight for a finisher pig in VETSTAT (Danish monitoring system for veterinary drug use at herd level [23]) is 50 kg [18] while the SDa uses a standardized weight of 70 kg for this category of animals. In this study the standardized weights of 303.8 kg and 70 kg were used in all analysis to avoid this problem, but when directly comparing results from national antimicrobial consumption reports this fact should be considered.

Also the level on which the antimicrobial consumption is calculated in the national monitoring has to be considered in comparisons. The differences between a national level calculation, where the mean use is weighted according to farm size, and a per farm analysis where all farms are of equal weight can be substantial. In the Netherlands the number of DDDA_NL_/Y is established both at national level and farm level (number of DDDA_F_/Y). The mean of the number of DDDA_F_/Y, however, is lower than the number of DDDA_NL_/Y (− 25.5 % for sows/piglet farms and −22.0 % for finisher pig farms for 2012) which indicates a higher use on large farms compared to small farms, at least within the pig sector. The calculation level (farm versus national) should therefore be verified before attempting across-country comparisons, at least when including data from the Netherlands.

## Conclusion

Our study revealed interesting differences in outcomes on antimicrobial consumption in pigs following the use of different animal defined daily dosages. Differences in outcomes on the use of AVMPs in an animal species can be attributed to the applied animal defined daily dosage due to differences in authorized indications and dosages, but can also be a result of differences in prescription patterns between farm types at the level of therapeutic groups or even specific AVMPs within those groups, next to the differences in animal (sub) categories and standardized animal weights.

This study underlines the urgent need for international harmonized units of measurement applicable in monitoring systems for antimicrobial use in livestock, such as generic animal defined daily dosages. But also harmonized animal (sub) categories and standardized animal weights for all animal species included in monitoring programs should be established, as proposed by the ESVAC.

## Abbreviations

ADD_DK_: Animal Daily Dosage as established in Denmark and applied previously
AVMP: Antimicrobial Veterinary Medicinal Product
DADD_DK_: Defined Animal Daily Dosage as currently applied in the Danish methodology
DANMAP: Danish Integrated Antimicrobial Resistance Monitoring and Research Program
DDD: Defined Daily Dosage (human)
DDDA: Defined daily dose animal
DDDA_NL_: Defined Daily Dose Animal as applied in the Dutch methodology
EAN: European Article Number
ESBL: Extended-spectrum beta-lactamase producing microorganism
ESVAC: European Surveillance of Veterinary Antimicrobial Consumption
SDa: The Netherlands Veterinary Medicines Authority
VETSTAT: The Danish veterinary monitoring system based on drug usage information collected at herd level
WHO: World Health Organization
